# Environmental Health Attitudes, Practices, and Educational Preferences: A National Survey of Reproductive-Aged Women in Canada

**DOI:** 10.3390/ijerph21111397

**Published:** 2024-10-23

**Authors:** Eric J. Crighton, Erica Phipps, Graeme N. Smith, Rukhsana Ahmed, Jocelynn L. Cook, Jeffrey R. Masuda, Alvaro R. Osornio-Vargas, Margaret Sanborn, Lesley J. Brennan, Karen P. Phillips, on behalf of the PEHE Collaboration

**Affiliations:** 1 Department of Geography, Environment and Geomatics, University of Ottawa, Ottawa, ON K1N 6N5, Canada; 2 Canadian Partnership for Children’s Health and Environment, Ottawa, ON K1S 2Z1, Canada; 3 Department of Obstetrics and Gynecology, Queen’s University, Kingston, ON K7L 2V7, Canada; gns@queensu.ca; 4 Department of Communication, University at Albany, State University of New York, Albany, NY 12222, USA; rahmed4@albany.edu; 5 Society of Obstetricians and Gynecologists of Canada, Ottawa, ON K1B 1A7, Canada; jcook@sogc.com; 6 School of Public Health and Social Policy, University of Victoria, Victoria, BC V8W 2Y2, Canada; jeffmasuda@uvic.ca; 7 Department of Pediatrics, University of Alberta, Edmonton, AB T6G 1C9, Canada; osornio@ualberta.ca (A.R.O.-V.); lbrennan@ualberta.ca (L.J.B.); 8 Department of Family Medicine, McMaster University, Hamilton, ON L8P 1H6, Canada; msanborn@bmts.com; 9 WHO Collaborating Centre in Child Health and the Environment, University of Alberta, Edmonton, AB T5R 4H5, Canada; 10 Interdisciplinary School of Health Sciences, University of Ottawa, Ottawa, ON K1N 6N5, Canada

**Keywords:** environmental health, toxic chemicals, pregnancy, prenatal education, reproductive health, child health, survey, Canada

## Abstract

Prenatal exposures to environmental toxicants can adversely affect fetal and child development and lead to increased risk of chronic disease. While regulatory action is essential to reduce sources of environmental toxicants, prenatal care presents an opportunity to educate, mobilize, and support prospective parents to reduce exposures to such hazards. As the first phase of an interdisciplinary research collaboration to inform the development of prenatal environmental health education strategy in Canada, we surveyed reproductive-aged female individuals. The online survey (July–September 2021) yielded a nationally representative sample of 1914 reproductive-aged females living in Canada. The questionnaire topics addressed the respondents’ knowledge and perceptions of environmental health risks, preventive actions and related facilitators and barriers, information sources and preferences, reproductive history, and demographics. The analysis included bivariate and multivariate techniques. Our results suggest broad awareness among reproductive-aged females that exposure to toxicants can be harmful, and that reducing prenatal exposures can benefit child health. However, fewer than half of respondents felt that they had enough knowledge to take protective measures. Despite high levels of preference for prenatal care as an ideal context for learning about environmental health risks and protective measures, fewer than one in four respondents had ever discussed environmental health concerns with a healthcare provider. Our findings reveal a knowledge–action gap and a corresponding opportunity to improve environmental health education and advocacy in prenatal care in the Canadian context.

## 1. Introduction

Prenatal exposures to common environmental toxicants, such as lead, pesticides, second-hand smoke, bisphenol A (BPA), flame retardants, air pollution, and workplace toxicants, can adversely affect fetal development and lead to negative reproductive and developmental health outcomes [1,2,3,4,5,6,7,8,9,10,11,12]. From conception through gestation, the developing fetus is particularly vulnerable to exogenous chemical exposures due to the dynamic and complex processes that occur as the brain and other organ systems develop, resulting in impaired development and lifelong health impacts [3,8,10]. Approximately 3% of fetal developmental defects are estimated to be attributable to chemical exposures, and an additional 25% to combinations of environmental and genetic factors [13,14]. Even at extremely low doses, exposures to certain common environmental chemicals during fetal development are risk factors for a range of neurodevelopmental impacts (e.g., intellectual or cognitive impairment linked to PCBs, polybrominated diphenyl ethers (PBDEs), and mercury) and chronic diseases such as asthma, diabetes, and certain cancers [3,15,16,17,18,19,20]. The presence of such risks can contribute to stress and mental health impacts, with associated implications for health and well-being, particularly among those who experience environmental health injustice and those affected by environmental disasters [21,22]. The COVID-19 pandemic, along with climate-related hazards such as wildfire smoke and extreme heat, is also recognized as contributing to negative impacts on health and well-being, with heightened susceptibility during pregnancy, fetal development, and childhood [23,24,25].

Biomonitoring studies in North America, Europe, and Asia indicate detectable levels of numerous toxic substances in the sera, urine, and cord blood taken from pregnant people, as well as in breastmilk [26,27,28,29,30,31,32,33,34,35,36,37]. Typical pregnancy-body burdens include chemicals recognized as carcinogens, neurotoxins, teratogens, and/or reproductive toxicants [38,39,40,41,42].

This growing evidence of toxicity combined with widespread population exposures underscores the need to reduce prenatal toxicant exposures. In addition to regulatory action, individuals need information and strategies to reduce their exposures at home, outdoors, and in work environments. Educational interventions have been found to increase the adoption of protective actions in the home [43] and in occupational contexts [44]. Problematically, while environmental risk information may be abundantly available (e.g., online), pregnant people remain insufficiently supported with clear information and guidance informed by the best available evidence. Of particular concern is a lack of proactive education and message reinforcement by perinatal care providers, as evidenced by an Ontario (Canada) study examining new mothers’ environmental risk perceptions and protective actions [45,46,47]. Furthermore, socioeconomic status, language, sense of control, and awareness were all important barriers to accessing information and taking protective action, highlighting the need for educational resources and strategies that are accessible and sensitive to individual and local community contexts [46,47]. A 2021 survey of reproductive-aged individuals in Australia, Canada, India, the United Kingdom, and the United States similarly reported lack of knowledge (23.8% within the control group) as the top barrier to taking protective action to reduce toxic exposures and the associated harms [48].

Developing effective and equitable environmental health promotion and preventive care strategies requires an integrated approach that recognizes the perspectives of the reproductive-aged public and their healthcare providers (HCPs), as well as the individual and structural factors that may promote or inhibit uptake of preventive care activities [49]. These factors include health beliefs and attitudes; educational resources; healthcare system characteristics; family and community resources and capacities, including income, housing, food security, and other social determinants of health; and the nature of the preventive action itself. Our understanding of these factors is critically lacking in Canada, representing a significant barrier to improving environmental health preventive care. To address this, we undertook an interdisciplinary Prenatal Environmental Health Education (PEHE) Collaboration research initiative to inform and catalyze the development of equity-focused and patient-centered education strategies for addressing toxicant exposures and other environmental health concerns as a routine part of preconception/prenatal care in Canada [50]. As the first phase of the PEHE initiative, we conducted a national survey of reproductive-aged female individuals designed to (1) investigate prenatal environmental health attitudes, protective practices, and educational preferences, and (2) identify individual and systemic factors that may influence their attitudes and practices. Although we recognize that other genders can conceive and gestate a pregnancy, the gender specifications in our study limited the survey participants to those identifying as female; as such, they are referred to throughout the manuscript as “women”.

## 2. Methods

### 2.1. Survey Design

Using a cross-sectional study design, we conducted an online survey guided by the Systems Model of Clinical Preventive Care [51] and informed by existing survey instruments [46,52]. The questionnaire topics (see Table 1) included (1) knowledge and perceptions about environmental health risks; (2) preventive actions taken and/or considered, and facilitators and barriers to taking preventive actions; (3) sources for information on health, environmental health, and pregnancy; (4) pregnancy history and personal experiences of infertility and adverse pregnancy or developmental outcomes; and (5) socioeconomic characteristics, geographic location, and demographic characteristics (Table 2). Four survey questions (see Table 1) informed the regression analysis described below. The survey question formats included Likert scales, yes/no/unsure, multiple-choice questions, and a small number of open-ended questions. The survey was pilot tested for comprehensibility, sensitivity, and response burden by 20 women, in both French and English. This research was approved by the University of Ottawa Research Ethics Board (File # S-02-21-6641).

**Table 1:** Survey questions used in the data analysis.

▪ Are there any actions you take to reduce your exposure to toxic chemicals or other environmental health hazards in your day-to-day life? * (Yes/No) ○ If yes: Please specify one or more actions: [Table 3] ▪ Are there any actions that you would like to take to protect your/ your family’s health from environmental hazards but are unable to? * (Yes/No) ○ If yes: Please specify the actions: [Table 4] ○ If no: What prevents you from taking those protective actions (Select all that apply): [Table 4] ▪ Generally speaking, would you say you know enough about toxic chemicals and other environmental hazards to take actions to protect yourself and/or your family? * (Yes, definitely/Yes, to some extent/No, not really/No, not at all) [Table 4] ▪ Did you ever discuss environmental hazards in your home, neighbourhood, school or workplace with a HCP (such as a family doctor, nurse, midwife, or public health professional)? * (Yes/No/Don’t know or don’t remember) [Table 5] ○ What type of healthcare provider? (Select all that apply) [Table 5] ▪ Have you ever felt reluctant or hesitant to ask a healthcare provider about your concerns about toxic chemicals or other environmental hazards? (Yes/No/Have not had concerns) ○ If yes: Why did you feel reluctant to ask a healthcare provider about your concerns about toxic chemicals or other hazards? Select all that apply. [Table 5] ▪ Have you ever heard or read about any environmental hazards that could harm a pregnancy or the health of the child to be born from the pregnancy? (Yes/No/Don’t know or don’t remember) [Table 5] ○ Please select the sources from which you heard or read about environmental hazards related to pregnancy. Select all that apply. [Table 5] ▪ When do you think environmental health hazards should be discussed? Select all that apply. [Table 5]

* Question used in regression analyses (Table 6 and Table 7).

### 2.2. Data Collection

Using the services of the Survey Research Centre at the University of Waterloo, the survey was conducted between 20 July and 13 September 2021, and we obtained a nationally representative sample of 1914 reproductive-aged female individuals (18–45 years) living in Canada. The participants were recruited from an existing Canadian national random digit dialing sample. An email invitation and up to two reminders were sent to panel members who satisfied the age (reproductive age—18 to 45 years), gender (female), and regional specifications (Canadian provinces). Respondents who agreed to participate were sent the online survey link in their preferred language, English or French. Responses to qualifying questions—namely, biological sex (females only), age (18–45 years only), and province/territory of residence—and responses regarding pregnancy status (never, current, past) and self-identification as Indigenous (yes, no), a visible minority (yes, no), or a newcomer to Canada within the past 10 years (yes, no) were required for survey completion. The participants were able to skip or decline other questions, as reflected in the data tables via the response counts. The Maritime Provinces (Nova Scotia, Newfoundland and Labrador, New Brunswick, and Prince Edward Island) were oversampled to ensure adequate statistical power, and the Territories (Northwest, Yukon, and Nunavut) were excluded from the sample due to recruitment limitations stemming from their small populations. Sample quotas were set based on age group, region, visible minority status, and Indigenous status in the overall sample to be representative of national levels. To account for unmet quotas, sampling weights were calculated using 2016 census data [53]. The small coefficient of variation (CV) in the weighted data (CV = 0.61) indicates that the unweighted data were already fairly representative prior to weighting.

### 2.3. Data Analysis

We analyzed the survey data using bivariate and multivariate techniques in SPSS v.28 (IBM Corp. New York, NY, USA). Our analysis involved the use of complex-sample descriptive and logistic regression procedures, which perform analysis on variables from samples drawn by complex sampling methods, as was the case here. For multivariate regression modeling, bivariate analysis was used to assess statistical significance between the dependent variables and potential independent predictor variables. We constructed four logistic regression models for the following dependent variables: (1) take day-to-day protective actions; (2) want to take protective actions but unable; (3) have enough knowledge to take protective actions; and (4) ever discussed hazards with HCP. Open-text responses were grouped into broad categories for inclusion in the analyses.

All significant independent variables identified in the bivariate analyses were entered into multivariate logistic regression models using a forward stepwise selection algorithm. Variables were determined to contribute to the model and retained if the significance level for the Wald inclusion test statistic was less than 0.05. Variables retained through the stepwise process were then entered into the final models. The variable “pregnancy status” was forced into each of the final models regardless of its contribution, owing to its a priori importance [54,55,56,57,58].

## 3. Results

### 3.1. Demographics

The demographic characteristics of the sample are shown in Table 2. Sample percentages were derived from weighted data, while counts were left unweighted to illustrate the sample distribution. Forty-one percent of the sample was under 30 years of age. Over half (54%) reported never having been pregnant, and just over 4% were currently pregnant at the time of data collection. Just over 5% reported being Indigenous, 28.6% visible minorities, and 9.6% recent immigrants. The sample percentages for age, Indigenous status, visible minority status, and geographic region are representative of the Canadian population. Approximately 87% of the sample reported having post-secondary education (e.g., some college or university education; graduate studies), 21% earned less than CAD 20,000 per year, and 15% reported being able to make ends meet only some of the time or never.

**Table 2:** Sample characteristics.

Characteristics	% (*n*) ^1^
Age, years	
18–29	41.1 (614)
30–39	37.1 (923)
40–45	21.8 (377)
Pregnancy history	
Never pregnant	54.1 (967)
Currently pregnant	4.4 (88)
Previously pregnant	41.5 (859)
Indigenous	5.2 (86)
Visible minority	28.6 (413)
Recent immigrant (<10 years ago)	9.6 (190)
Education	
High school or less	13.3 (247)
College or university	73.8 (1429)
Graduate studies	12.9 (236)
Household gross annual income (CAD)	
<40,000	21.1 (392)
40,000 to <60,000	16.1 (304)
60,000 to <100,000	31.8 (618)
≥100,000	30.9 (589)
Able to make ends meet	
All or most of the time	84.9 (1595)
Some of the time to never	15.2 (314)
Geographic region/province	
British Columbia	13.2 (269)
Prairies ^2^	19.4 (403)
Ontario	38.9 (689)
Quebec	22.5 (359)
Maritimes ^3^	6.0 (194)
Community type	
Big city	37.1 (683)
Suburbs of big city	31.8 (581)
Town or small city	24.0 (492)
Village or rural area	7.1 (155)

^1^ Percentages derived from weighted data, *n* from unweighted data. ^2^ Prairies include the Canadian provinces of Alberta, Saskatchewan, and Manitoba. ^3^ Maritimes include the eastern provinces of New Brunswick, Nova Scotia, Prince Edward Island, and Newfoundland and Labrador.

### 3.2. Perceptions of Environmental Risk to Pregnancy and Child Health

Among the respondents, most (91.9%) reported that they agreed or strongly agreed that day-to-day exposures to toxic chemicals in the womb and during early childhood can be harmful to children’s development and long-term health (Table 3), and over half (54.5%) reported being moderately to very concerned about these hazards. When asked to identify hazards of concern, those most commonly mentioned were toxic chemicals (23.5%) and air pollution (14.2%). The toxic chemicals category (23.5%) under-represents the overall level of concern about toxic substances, which was also reflected in pesticides (6.6%), metals (5.6%), and in responses related to pollution, workplace concerns, plastics, and food and water quality.

**Table 3:** Concern regarding the impacts of environmental hazards on prenatal and child health.

Environmental Concern Variables	Responses % (#) ^1^
Day-to-day exposures can be harmful to child health (*n* = 1855) ^1^	
Agree to strongly agree	91.9 (1704)
Strongly disagree to neutral	8.1 (151)
Level of concern about exposure to environmental hazards during pregnancy and potential effects on personal or child health (*n* = 1859) ^1^	
Moderately to very concerned	54.5 (990)
Not at all to slightly concerned	45.5 (869)
Hazards of concern (*n* = 2912) ^2^	
Toxic chemicals ^3^	23.5 (683)
Air pollution	14.2 (413)
Pollution—general	8.4 (246)
Climate change/natural disasters/wildfire smoke	7.0 (205)
Second-hand smoke	6.8 (197)
Pesticides	6.6 (191)
Food exposures	6.0 (176)
Metals (e.g., lead, mercury)	5.6 (162)
Pathogens—disease	4.2 (121)
Drinking water quality	4.1 (120)
Radiation	2.9 (84)
Plastics	2.4 (69)
Workplace exposures	1.6 (46)
Pharmaceuticals	1.3 (37)
Mold	1.2 (36)
Other ^4^	4.3 (126)

^1^ Percentages derived from weighted data, *n* from unweighted data. ^2^ *n* is greater than the sample size due to the possibility of multiple responses to this question. ^3^ Includes general responses (e.g., “toxic chemicals”) and specific substances (e.g., “BPA”, “phthalates”) but excludes metals and pesticides, which were categorized separately. Respondents’ concerns categorized into workplace exposures, pollution, food exposures, drinking water quality, and/or plastics may also reflect concern about toxic chemicals. As such, 23.5% understates the overall concern about toxic chemicals. ^4^ Examples of “other” reported concerns include alcohol, acid rain, stress, nicotine, and noise pollution.

### 3.3. Environmental Risk Mitigation Actions and Barriers

Most of the respondents (90.6%) indicated that they agreed or strongly agreed that pregnant people can increase their chances of having a healthy child by reducing their exposure to toxic chemicals and other environmental hazards, while just over half (52.2%) indicated that they definitely or to some extent had enough knowledge to take actions to protect themselves and/or their families (Table 4). Over half of the sample (56.2%) said that they took day-to-day preventative actions to protect their own health or the health of their family, including opting for less/non-toxic and natural everyday products (32.2%), avoiding/reducing exposure to pollutants/toxicants (21.6%), and other actions (Table 4). Just under 40% indicated that they wanted to take protective actions but were unable, with reported barriers including costs (52.7%), being unaware of safer options (39.5%), lack of time (22.3%), lack of partner support (11.3%), and lack of employer support (6.9%). One in five (19.8%) survey respondents reported that the COVID-19 pandemic further prevented them from taking action to avoid toxic chemicals or other environmental hazards in their day-to-day lives. Among the COVID-19-related barriers to risk mitigation were spending more time at home and having less money available.

**Table 4:** Preventive actions taken and/or considered, and barriers to action.

Preventive Action Variables	Responses % (#) ^1^
Pregnant persons can reduce risk by reducing exposures (*n* = 1839)	
Agree to strongly agree	90.6 (1661)
Strongly disagree to neutral	9.4 (178)
Have enough knowledge to take protective actions (*n* = 1913)	
Definitely or to some extent	52.2 (1026)
Not really or not at all	47.8 (887)
Take protective actions in day-to-day life (*n* = 1911)	56.2 (1097)
Most commonly reported protective actions (*n* = 1729) ^2^	
Opting for less/non-toxic and natural everyday products	32.2 (556)
Avoiding/reducing exposure to pollutants/toxicants	21.6 (373)
Choosing healthy/toxicant-free food	16.7 (288)
Using masks and other PPE ^3^	6.4 (110)
Acting to reduce personal environmental impact	4.3 (74)
Trying to be better informed	4.0 (69)
Changing workplace/living arrangements/daily practices	4.0 (69)
Using safer plastics/avoiding plastics	3.9 (67)
Taking steps to improve the quality of drinking water	3.2 (55)
Other	3.9 (68)
Want to take protective actions but unable (*n* = 1910)	39.6 (754)
Barriers to taking protective action (*n* = 750)	
Costs	52.7 (395)
Unaware of safer options	39.5 (296)
Time	22.3 (167)
Lack of partner support	11.3 (85)
Exposure is out of my control	9.7 (73)
Lack of employer support	6.9 (52)
Lack of landlord support	6.5 (49)
Action will do more harm	4.0 (30)
Other ^4^	1.7 (13)

^1^ Note: Percentages derived from weighted data, *n* from unweighted data. ^2^ *n* is greater than the *n* for this question due to the possibility of multiple responses. ^3^ PPE: personal protective equipment. ^4^ “Other” reported barriers to taking action include lack of government support, vaccination mandates, and lack of support from neighbors.

### 3.4. Sources of Environmental Health Information

Less than half (42.8%) of the sample reported that they had at some point heard or read about environmental hazards that could harm a pregnancy or the health of the child to be born from the pregnancy, with the most common sources of information being the Internet (62.1%), news media (56.4%), family or friends (45.8%), HCPs (43.7%), and social media (36.1%; Table 5). Of those who had received information, most (89.4%) reported that it did or would influence their action during pregnancy somewhat or a lot. Only 22.7% indicated that they had ever discussed concerns about environmental hazards with their HCP, with one-fifth of respondents (19.9%) sharing that they were reluctant to ask their HCP about environmental hazards. Reported reasons for this reluctance included the perception that the HCP might dismiss their concerns (65.8%), not have the information (42.8%), or not have the time (40.3%). Other reported reasons included participants’ perceptions that their question might not be valid (35.2%), or that they did not have enough knowledge to know what they should be asking (35.0%). When asked when environmental hazards related to pregnancy should be discussed, pregnancy-related healthcare was most commonly cited (77.1%), followed by routine well-baby/well-child healthcare (56.6%) and routine adult healthcare (53.4%). Half of the respondents (49.6%) indicated that the topic should also be part of the high school health curriculum. A small minority (3.5%) felt that this topic does not need to be discussed.

**Table 5:** Sources for information on environmental health and pregnancy.

Environmental Health Information Variables	Responses % (*n*) ^1^
Heard/read about environmental hazards harmful to pregnancy/child (*n* = 1914)	42.8 (823)
Source of information about environmental hazards (*n* = 823)	
Internet	62.1 (509)
News media	56.4 (454)
Family members/friends	45.8 (378)
Doctor, nurse, midwife, or other provider	43.7 (381)
Social media	36.1 (301)
Public health professional	32.9 (281)
Health-related books or magazines	28.5 (242)
Other ^2^	5.4 (42)
Info did/would influence action during pregnancy (*n* = 822)	
Somewhat to a lot	89.4 (729)
Not at all to a little	10.6 (93)
Ever discussed environmental hazards with healthcare provider (*n* = 1912)	22.7 (445)
Reluctant to ask healthcare provider about environmental hazards (*n* = 1570)	19.9 (295)
Reason for reluctance	
Healthcare provider might dismiss the concerns	65.8 (186)
Healthcare provider might not have the information	42.8 (130)
Healthcare provider might not have time	40.3 (117)
Not sure question/concern was valid	35.2 (107)
Did not have enough knowledge to know what to ask healthcare provider	35.0 (111)
Had more important questions to ask	29.8 (92)
Would not have trusted healthcare provider’s advice	6.8 (24)
Context for environmental health education (*n* = 1914)	
Pregnancy-related healthcare	77.1 (1477)
Routine well-baby/well-child healthcare	56.6 (1098)
Routine adult healthcare	53.4 (1010)
High school health curriculum	49.6 (931)

^1^ Percentages derived from weighted data, *n* from unweighted data. ^2^ Other reported sources of information about environmental hazards include university, high school, and work training.

### 3.5. Factors Informing Environmental Mitigation Strategies: Actions and Barriers

Table 6 shows the results of two multivariate logistic regression models: (1) taking day-to-day protective actions, and (2) wanting to take protective actions but being unable. The analysis indicates that respondents were more likely to report taking day-to-day protective actions if they had a past pregnancy (OR: 1.43; CI = 1.07–1.90), were in the 30–39-year age group (OR:1.86; CI = 1.37–2.53), were moderately to very concerned that environmental exposures might harm their health or the health of their child (OR: 2.47; CI = 1.89–3.23), agreed or strongly agreed that day-to-day environmental exposures can be harmful to children’s health (OR: 2.63; CI = 1.60–4.32), had enough knowledge to take protective actions (OR: 2.35; CI = 1.80–3.08), and had ever discussed hazards with an HCP (OR: 2.22; CI = 1.60–3.08). Respondents who wanted to take (other) protective actions but were unable were still likely to take daily protective actions (OR: 1.85; CI = 1.39–2.45). Respondents were more likely to report wanting to take actions but unable if they were in the oldest age group (40–45 years; OR: 1.81; CI = 1.25–2.64), were moderately to very concerned that environmental exposures might harm their health or the health of their child (OR: 2.37; CI = 1.80–3.09), were already taking day-to-day protective actions (OR: 2.04; CI = 1.54–2.69), and had ever discussed hazards with an HCP (OR: 1.77; CI = 1.33–2.36).

**Table 6:** Factors affecting the likelihood of taking day-to-day protective actions, and of wanting but being unable to take proactive actions, using multivariate logistic regression analyses.

Variables	Taking Day-to-Day Protective Actions	Wanting to Take Protective Actions but Unable
	OR (95% CI)	OR (95% CI)
Pregnancy status		
Never	1.0	1.0
Currently	1.63 (0.89–2.97)	0.82 (0.42–0.1.61)
Past	1.43 (1.07–1.90) *	0.97 (0.74–1.27)
Age group, years		
18–29	1.0	1.0
30–39	1.86 (1.37–2.53) ***	1.18 (0.87–1.58)
40–45	1.32 (0.90–1.93)	1.81 (1.25–2.64) **
Level of concern about environmental exposures		
Not at all to slightly	1.0	1.0
Moderately to very	2.47 (1.89–3.23) ***	2.37(1.80–3.09) ***
Day-to-day exposures can be harmful to child health		
Strongly disagree to neutral	1.0	
Agree to strongly agree	2.63 (1.60–4.32) ***	
Take day-to-day protective actions		
No		1.0
Yes		2.04 (1.54–2.69) ***
Want to take protective actions but unable ^1^		
No	1.0	
Yes	1.85 (1.39–2.45) ***	
Enough knowledge to take protective actions		
Not really to not at all	1.0	
To some extent or definitely	2.35 (1.80–3.08) ***	
Ever discussed hazards with healthcare provider		
No	1.0	1.0
Yes	2.22 (1.60–3.08) ***	1.77 (1.33–2.36) ***
Observations (unweighted)	*n* = 1556	*n* = 1598
Pseudo R^2^ (Nagelkerke)	0.274	0.158

Note: OR = odds ratio, 95% CI = (95% confidence interval); * *p* < 0.05, ** *p* < 0.01, *** *p* < 0.001. ^1^ Respondents, even those who reported taking protective actions, were asked if there were actions that they would like to take to protect their own health or their family’s health from environmental hazards but were unable to.

Table 7 shows the results of two multivariate logistic regression models: (1) enough knowledge to take protective actions, and (2) ever discussed hazards with HCP. Respondents were more likely to report having enough knowledge to take protective actions if they had previously been pregnant (OR:1.58; CI = 1.26–1.99), agreed or strongly agreed that day-to-day exposures can be harmful to child health (OR: 1.59; CI = 1.10–2.31), took day-to-day protective actions (OR:2.61; CI = 2.06–3.31), and had ever discussed hazards with a HCP (OR: 2.05; CI = 1.52–2.76). Respondents were found to be more likely to report ever having discussed hazards with an HCP if they were currently pregnant (OR: 2.29; CI = 1.33–3.94) or previously pregnant (OR: 1.72; CI = 1.29–2.30), had immigrated to Canada within the past 10 years (OR: 2.91; CI = 1.94–4.39), were moderately to very concerned that environmental exposures might harm their health or the health of their child (OR: 1.60; CI = 1.15–2.22), took day-to-day protective actions (OR: 2.15; CI = 1.57–2.95), wanted to take protective actions but were unable (OR: 1.62; CI = 1.23–2.14), and reported to some extent or definitely having enough knowledge to take protective actions (OR: 1.94; CI = 1.45–2.61).

**Table 7:** Factors affecting the likelihood of reporting enough knowledge to take protective actions, and of ever having discussed concerns about environmental health risks with a healthcare provider, using multivariate logistic regression analyses.

Variables	Enough Knowledge to Take Protective Actions	Ever Discussed Hazards with Healthcare Provider
	OR (95% CI)	OR (95% CI)
Pregnancy status		
Never	1.0	1.0
Currently	1.46 (0.88–2.42)	2.29 (1.33–3.94) **
Past	1.58 (1.26–1.99) ***	1.72 (1.29–2.30) ***
Immigrated (within last 10 years)		
No		1.0
Yes		2.91 (1.94–4.39) ***
Level of concern about environmental exposures		
Not at all to slightly		1.0
Moderately to very		1.60 (1.15–2.22) **
Perceptions that day-to-day exposures can be harmful to child health		
Strongly disagree to neutral	1.0	
Agree to strongly agree	1.59 (1.10–2.31) *	
Take day-to-day protective actions		
No	1.0	1.0
Yes	2.61 (2.06–3.31) ***	2.15 (1.57–2.95) ***
Want to take protective actions but unable		
No		1.0
Yes		1.62 (1.23–2.14) ***
Enough knowledge to take protective actions		
Not really to not at all		1.0
To some extent or definitely		1.94 (1.45–2.61) ***
Ever discussed hazards with healthcare provider		
No	1.0	
Yes	2.05 (1.52–2.76) ***	
Observations (unweighted)	*n* = 1849	*n* = 1848
Pseudo R^2^ (Nagelkerke)	0.148	0.189

Note: OR = odds ratio, 95% CI = (95% confidence interval); * *p* < 0.05, ** *p* < 0.01, *** *p* < 0.001.

## 4. Discussion

The results of a national survey of reproductive-aged women’s attitudes, protective practices, and educational preferences related to prenatal environmental health point to an important opportunity to address a knowledge–action gap through enhanced education and advocacy. This study—conducted as the first phase of an intersectoral initiative to inform prenatal environmental health education strategies in Canada—revealed broad awareness of the potential harms to child health associated with prenatal and early-life exposures to toxic chemicals, along with similarly high levels of agreement that taking action to reduce adverse environmental exposures during pregnancy can reduce risks to child health. However, nearly half of respondents reported being only somewhat or not at all concerned about environmental hazards related to a past/current/potential pregnancy. Only about half reported taking protective measures in their day-to-day life to protect themselves or their family from environmental health hazards, similar to levels found elsewhere [59]. Multiple barriers to action were identified, including those that underscore underlying economic and structural inequities, such as the cost of safer options and lack of support from employers and landlords. Insufficient practical knowledge emerged as an important and actionable barrier. Fewer than half (47.8%) reported having enough knowledge to take actions to protect themselves and/or their families from environmental hazards. The survey’s findings further suggest an appetite for prenatal environmental health education, with nearly three out of four respondents identifying prenatal care as the preferred context for such learning, despite the fact that fewer than one in four reported having had such conversations with their HCP.

### 4.1. Perceptions of Environmental Health Risks

The differences in levels of awareness and concern among respondents about the effects of toxic chemicals and other environmental hazards during their own current/past/future pregnancy (just over half, 54%), as compared to the more generalized awareness about the potential effects of such hazards on child health (92% agreement), underscore the underdeveloped status of prenatal environmental health education in Canada. This difference may suggest a greater degree of societal awareness of children’s environmental health risks and less familiarity with the “hidden” risks that can occur during pregnancy. Unlike other prenatal counseling topics, such as alcohol and smoking, the respondents in our study were not routinely having conversations with their HCPs about environmental exposures.

Toxic chemicals emerged as the most frequently cited environmental health concern in the context of pregnancy. The rise in the prevalence of children’s developmental disorders, including autism, is commonly attributed to gestational and/or childhood exposure to environmental hazards by parents [60]. Respondents’ concerns about toxic chemicals also reflect growing public awareness and concern (and related marketing) related to chemicals such as bisphenol-A (BPA), phthalates, and the per- and polyfluoroalkyl substances (PFASs), commonly referred to as “forever chemicals”. The developmental risks of climate change and natural disasters are often less direct, manifesting as exposures to extreme heat, wildfire smoke, flooding, and potential displacement, as well as societal disruptions to health services, all of which disproportionately affect populations marginalized by poverty, racism, colonialism, and housing instability [61,62]. While only 7% of respondents identified climate change and natural disasters as a concern in the context of pregnancy due to potential harm to their own health or that of the expected child, it is worth noting that this level of concern exceeded that reported for well-established environmental risks, including pesticides (6.6%) and metals such as lead and mercury (5.6%).

### 4.2. Environmental Health Literacy as Foundational to Individual and Collective Action

The survey results highlight a link between existing knowledge and inclination towards action. Respondents with higher self-reported levels of environmental health knowledge were more likely to report taking protective measures. This is consistent with the large body of risk perception literature in which high educational status, often associated with higher socioeconomic privilege, is equated with an increased locus of control to reduce environmental exposures through behavioral strategies [63,64,65]. Our findings also indicate that respondents with greater environmental knowledge were also more likely to initiate a conversation with their HCP about environmental health concerns. Educational attainment—here, self-reported environmental knowledge—improves patients’ self-efficacy and ability to advocate for their health needs [66,67]. This suggests the need to focus resources on prospective parents who are not already well equipped with environmental health knowledge and related capacities. Respondents’ hesitancy and perceived barriers to raising concerns with their HCP about environmental health concerns also point to opportunities to improve patient–provider interaction. Supporting perinatal care providers in creating an atmosphere of receptivity to environmental health discussions would likely necessitate greater investments in HCP education (e.g., professional training, continuing education) and increased availability of and access to informational resources for patients.

The connection between existing awareness and interest in learning more is relevant to future educational strategies. Efforts to build environmental health literacy among the general population may provide an essential foundation for the uptake of environmental health education efforts in the context of prenatal care. Environmental literacy must include a basic ability to “recognize” the potential for an environmental exposure to be risky, and to take steps to reduce/avoid exposure or mitigate the risk [68,69]. Strategies to inform the public about the nature of developmental/childhood environmental risks must strive to convey the current state of the science, scientific uncertainties, and risk mitigation strategies, supported by the precautionary principle [70,71]. Embedded within models of environmental health literacy are concepts of environmental justice that recognize the disproportionate environmental risks faced by communities marginalized by racialization, poverty, and other social risk factors [68]. Promoting environmental health literacy supports the mobilization of citizen science, academic–community partnerships, and public demand for policies and investments to address environmental health disparities [68].

### 4.3. Environmental Health Information in the Context of Prenatal Care

Respondents reported learning about environmental risks to pregnancy/child health from a range of sources (e.g., Internet, news media, friends and family, HCPs), consistent with previous risk perception studies [45,65,72]. Importantly, such information was identified as influencing actions. In practical terms, this means that there are widespread knowledge mobilization pathways for equipping prospective parents with information on protective practices, and that such educational efforts would likely translate into increased action. A study in France to assess women’s knowledge, attitudes, and behaviors related to endocrine-disrupting chemicals and pregnancy similarly found interest in taking action to reduce exposures and mitigate health risks [73].

Our findings point to prenatal care as the preferred context for women to learn about environmental health risks to reproductive/child health and ways to prevent/reduce exposures, consistent with the findings of other studies. A qualitative study in Ontario, Canada on pregnant women’s navigation of environmental chemical risks revealed a preference among participants for “strong” information sources such as healthcare providers, professional bodies, and government, as well as “unified” risk messaging that is consistent across multiple strong sources [72]. While perinatal health providers often recognize the value of environmental health counseling and are well positioned to provide individualized guidance to help patients adopt mitigation strategies, studies have shown that scientific uncertainties, lack of environmental health training, and other barriers contribute to the clinical practice gap that is mirrored in our findings [74,75,76]. As such, any strategy to improve environmental health knowledge and agency among prospective parents must be paired with a parallel strategy to build the requisite capacity among HCPs. As a step in this direction, the PEHE Collaboration’s parallel survey of prenatal care providers in Canada seeks to contribute a better understanding of the experiences, barriers, and capacities of prenatal care providers from various disciplines (e.g., physicians, nurses, midwives, public health professionals) to engage with prospective parents on environmental health exposures and protective strategies.

Given the widespread (yet not universal) status of HCPs as trusted sources of health-related information [76], our findings suggest an important role for prenatal HCPs in educating and supporting prospective parents on environmental health issues as a routine part of prenatal care, with corresponding attention to the need to build trust and foster non-judgmental and supportive interactions with individuals and communities that experience socioeconomic marginalization and environmental injustice. The role of HCPs to engage in prenatal environmental health education and advocacy is already endorsed by professional associations in the United States [77], the United Kingdom [78], and internationally [79]. While also proposed here in Canada [80], our findings suggest that prenatal environmental health education remains underdeveloped in Canada.

### 4.4. Limitations

Limitations of this study include its timing vis-à-vis the COVID-19 pandemic, the use of an existing panel of online respondents, and insufficient representation of relevant populations, including residents of the Territories, marginalized populations, populations with low educational attainment, and prospective parents who identify as other than female, including fathers. Furthermore, the lack of geographic data to support the contextualizing of participants’ responses, along with the possibility that the use of words such as “toxic” and “hazard” predisposed respondents to register concern, also presented limitations. Firstly, this study was conducted during the COVID-19 pandemic. Initially scheduled for July 2020, the survey was postponed due to the concern that fear of COVID-19 would dominate the participants’ thoughts. Although the survey was delayed until the following summer (2021) to minimize the effects of COVID-19, concerns about the virus and associated protective measures were commonly reported. Despite this, the findings here regarding overall levels of concern about environmental health issues were similar to those of an earlier, pre-pandemic Ontario study [46]. A second potential limitation stems from the use of a panel sample, as described by [81]. Thirdly, due to the limitations of the panel sample, women of reproductive age living in Nunavut, Yukon, and the Northwest Territories were not included, an omission that warrants follow-up. The small sample size of racialized, newcomer, and Indigenous respondents likely constrained the ability of this study to reveal important dimensions of environmental health concerns and HCP–patient interaction within equity-denied populations, which warrants further research and action. Similarly, our sampling frame was skewed towards respondents with post-secondary education; thus, our findings inadequately reflect the likely role of education in mediating perceptions of environmental health risks and HCP–patient interaction. As such, they cannot be generalized to the Canadian population. Furthermore, the lack of granular geographic data (e.g., postal codes) constrained our ability to examine the respondents’ perceptions and experiences in the context of environmental justice concerns, such as proximity to pollution sources. The survey was also limited to females, despite growing scientific knowledge of the relevance of paternal environmental exposures and the potential role of partners in supporting/constraining pregnant persons’ efforts to reduce/avoid harmful exposures. Finally, we acknowledge that our decision to use terms commonly used in societal discourse about environmental health risks (e.g., “toxic”) for purposes of clarity and transparency may have increased the respondents’ propensity to report levels of concern in accordance with social norms, such as the protection of children.

## 5. Conclusions

Overall, our results demonstrate significant concern about environmental health risks among women of reproductive age, a corresponding motivation to take protective action, and a desire for information and education, particularly in the context of prenatal care. As such, this study can inform much-needed efforts to develop practicable and effective prenatal environmental health education practices in Canada. Additional barriers identified by the respondents that inhibit the adoption of protective practices (e.g., cost; lack of support from partners, employers, and landlords) underscore the importance of locally contextualized strategies that integrate action on the social determinants of health inequity, such as economic disparities, unfit housing, and uneven workplace protections.

The findings from this survey will be integrated with results from the PEHE Collaboration’s survey of prenatal care providers to inform the development of environmental health education strategies toward the goal of advancing an important yet underserved component of reproductive and child health protection in Canada.

## Data Availability

Survey response data relevant to this study are included as data tables. Open-ended survey responses that support the findings of this study are available from the corresponding authors (E.P./K.P.P.) upon request, but restrictions apply to the dissemination of these data.
